# Recurrent Neural Networks in Computer-Based Clinical Decision Support for Laryngopathies: An Experimental Study

**DOI:** 10.1155/2011/289398

**Published:** 2011-10-04

**Authors:** Jarosław Szkoła, Krzysztof Pancerz, Jan Warchoł

**Affiliations:** ^1^Institute of Biomedical Informatics, University of Information Technology and Management, Sucharskiego Str. 2, 35-225 Rzeszów, Poland; ^2^Department of Biophysics, Medical University of Lublin, Jaczewskiego Str. 4, 20-090 Lublin, Poland

## Abstract

The main goal of this paper is to give the basis for creating a computer-based clinical decision support (CDS) system for laryngopathies. One of approaches which can be used in the proposed CDS is based on the speech signal analysis using recurrent neural networks (RNNs). RNNs can be used for pattern recognition in time series data due to their ability of memorizing some information from the past. The Elman networks (ENs) are a classical representative of RNNs. To improve learning ability of ENs, we may modify and combine them with another kind of RNNs, namely, with the Jordan networks. The modified Elman-Jordan networks (EJNs) manifest a faster and more exact achievement of the target pattern. Validation experiments were carried out on speech signals of patients from the control group and with two kinds of laryngopathies.

## 1. Introduction

Computer-based clinical decision support (CDS) is defined as the use of a computer to bring relevant knowledge to bear on the health care and well-being of a patient [1]. Our research concerns with designing methods for CDS in a noninvasive diagnosis of selected larynx diseases. Two diseases are taken into consideration: Reinke's edema (RE) and laryngeal polyp (LP). In general, the diagnosis is based on an intelligent analysis of selected parameters of a patient's speech signal (phonation). The proposed approach is noninvasive. Comparing it to direct methods shows that it has several advantages. It is convenient for the patient because a measurement instrument (in this case, a microphone) is located outside the voice organ. This enables free articulation. Moreover, different physiological and psychological patient factors impede making a diagnosis using direct methods. From the clinical point of view, an early diagnosis enables taking an effective treatment without surgical procedures. The problem of larynx diseases has become an increasingly serious health problem in different occupational groups.

The majority of methods proposed to date are based only on the statistical analysis of the speech spectrum (e.g., [2]) as well as the wavelet analysis. An application of such methods does not always adjudicate the patient classification in a unique way. Our plan is to propose a hybrid approach, which is additionally based on a signal analysis in the time domain. Preliminary observations of signal samples for patients from a control group and patients with a confirmed pathology clearly indicate deformations of standard articulation in precise time intervals. In the paper, we propose an approach to the signal analysis in the time domain using recurrent neural networks (RNNs), especially, the Elman and Jordan networks [3, 4] also known as “simple recurrent networks.”

Our research concerns designing effective methods for computer support of a noninvasive diagnosis of selected larynx diseases. There exist various approaches to analysis of biomedical signals (cf. [5]). In general, we can distinguish three groups of methods according to a domain of the signal analysis: analysis in a time domain, analysis in a frequency domain (spectrum analysis), and analysis in a time-frequency domain (e.g., wavelet analysis). Therefore, in our research, we are going to build a specialized computer tool for supporting diagnosis of laryngopathies based on a hybrid approach. One part of this tool, playing an important role in a preliminary stage, will be based on the patients' speech signal analysis in the time domain. Hybridization means that a decision support system will have a hierarchical structure based on multiple classifiers working on signals in time and frequency domains.

A series of papers published earlier (see [2, 6–8]) has presented approaches leading to the approach shown in this paper. In this approach, designing the way of recognition of temporal patterns and their replications becomes the key element. It enables detecting all nonnatural disturbances in articulation of selected phonemes. For the time domain analysis, we propose to use neural networks with the capability of extracting the phoneme articulation pattern for a given patient (articulation is an individual patient feature) and the capability of assessment of its replication in the whole examined signal. Preliminary observations show that significant replication disturbances in time appear for patients with the clinical diagnosis of disease.

The capabilities mentioned are possessed by recurrent neural networks. One class of them are the Elman neural networks (ENs) [3]. In real-time decision making, an important thing is to speed up a learning process for neural networks. Moreover, accuracy of learning of signal patterns plays an important role. Therefore, in this paper, we propose some improvement of learning ability of ENs by combining them with another kind of RNNs, namely, the Jordan networks [4], and by providing some additional modification. A new resulting kind of RNNs is called the modified Elman-Jordan networks (EJNs).

The remaining part of the paper is organized as follows. After the introduction, we shortly describe the medical background related to larynx diseases (Section 2). In Section 3, we indicate basic problems in examination of a speech organ function for medical diagnosis.  Section 4 describes a structure and features of the modified Elman-Jordan neural network used for supporting diagnosis of laryngopathies. In Section 5, we present results obtained by experiments done on real-life data. Some conclusions and final remarks are given in Section 6.

## 2. Medical Background

A model of speech generation is based on the “source–filter” combination. The source is larynx stimulation, that is, passive vibration of the vocal folds as a result of an increased subglottis pressure. Such a phenomenon of making speech sonorous in the glottis space is called phonation. The filter is the remaining articulators of the speech canal creating resonance spaces. A signal of larynx stimulation is shaped and modulated in these spaces. A final product of this process is called speech.

Pathological changes appearing in the glottis space entail a bigger or smaller impairment of the phonation functions of the larynx. The subject matter of presented research concerns with diseases, which appear on the vocal folds, that is, they have a direct influence on phonation [9].

We are interested in two diseases: Reinke's edema (*Oedema Reinke*) and laryngeal polyp (*Polypus laryngis*).

### 2.1. Reinke's Edema

Reinke's edema appears often bilaterally and usually asymmetrically on the vocal folds. It is created by transudation in a slotted epithelial space of folds devoid of lymphatic vessels and glands, called the Reinke's space. In the pathogenesis of disease, a big role is played by irritation of the laryngeal mucosa by different factors like smoking, excessive vocal effort, inhalatory toxins, or allergens. The main symptoms are the following: hoarseness resulting from disturbance of vocal fold vibration or, in the case of large edemas, inspiratory dyspnea. In the case of Reinke's edemas, conservative therapy is not applied. They are microsurgically removed by decortication with saving the vocal muscle.

### 2.2. Laryngeal Polyp

Laryngeal polyp is a benign tumor arising as a result of gentle hyperplasia of fibrous tissue in mucous membrane of the vocal folds. In the pathogenesis, a big role is played by factors causing chronic larynx inflammation and irritation of the mucous membranes of the vocal folds: smoking, excessive vocal effort, reflux, and so forth. The main symptoms are the following: hoarseness, aphonia, cough, and tickling in the larynx. In the case of very big polyps, dyspnea may appear. However, not-big polyps may be confused with vocal tumors especially when there is a factor of the load of the patient voice. The polyp may be pedunculated or may be placed on the wide base. If it is necessary, polyps are microsurgically removed with saving a free edge of vocal fold and vocal muscle.

## 3. Basic Problems in Assessment of Voice

The research proves that a subjective assessment of voice is always reflected in the basic acoustic parameters of a speech signal. Sound parameters correlated with the anatomical structure and functional features of the voice organ are a subject of interest for researchers. However, the diversity of anatomical forms, inborn phonation habits, and the diversity of an exploratory material cause that researches are performed on different grounds. The voice generation is conditioned by a lot of factors, which give that voice an individual, peculiar character. However, the analysis of individual features of a speech signal in an appropriate group of people, suitably numerous, shows some convergence to values of tested parameters. This enables differentiation of changes of characteristics of the source (larynx stimulation) caused by different pathologies. Since a colloquial speech is a stochastic process, an exploratory material is often made up of vowels uttered separately with extended articulation. Together with the lack of intonation, it enables eliminating phonation habits.

We can distinguish two types of the acoustic measurement methods: objective and subjective. Both of them belong to indirect exploratory methods. Comparing them to direct methods (e.g., computer roentgenography, stroboscopy, bioelectrical systems) shows that they have several advantages. They are convenient for a patient because a measurement instrument (in this case, a microphone) is located outside the voice organ. This enables free articulation. The advantage of acoustic methods is the possibility of automating measurements using a computer technique. It is also possible to visualize individual parameters of a speech signal. Subjective auscultatory methods are used, among others, in laryngology and phoniatrics in case of both correct or pathological voice emission. Objective methods are based on physical features of the voice. They become especially popular, when a computer technique reaches a high extent of specialization. They enable the objective assessment of voice and deliver information in case of pathology and rehabilitation of the voice organ. Examined parameters aid the doctor's assessment of the patient's health state.

In the literature, we may notice that parameters of the source (larynx stimulation) are often examined, for example, [10]. However, it is possible to modify an exploratory method so that it encompasses wider range of the material analyzed. A crucial role is played by further mathematical processing of basic acoustic parameters. In this way, we can take into consideration and examine dynamic changes during the phonation process resulting from functions of the speech apparatus as well as from additional acoustic effects occurring in the whole voice organ.

## 4. Recurrent Neural Networks in Indicating Deformations of Articulation

In most cases, neural network topologies can be divided into two broad categories: feedforward (with no loops and connections within the same layer) and recurrent (with possible feedback loops). The Hopfield network, the Elman network, and the Jordan network are the best known recurrent networks. In the paper, we are interested in the two last ones.

**Figure 1:**
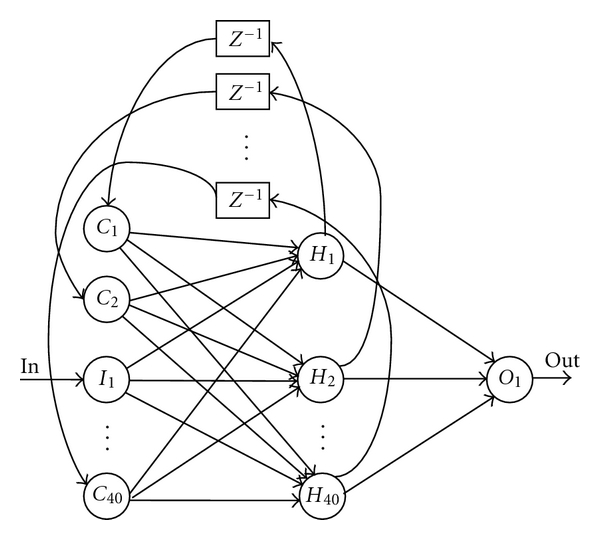
A structure of the trained Elman neural network.

**Figure 2:**
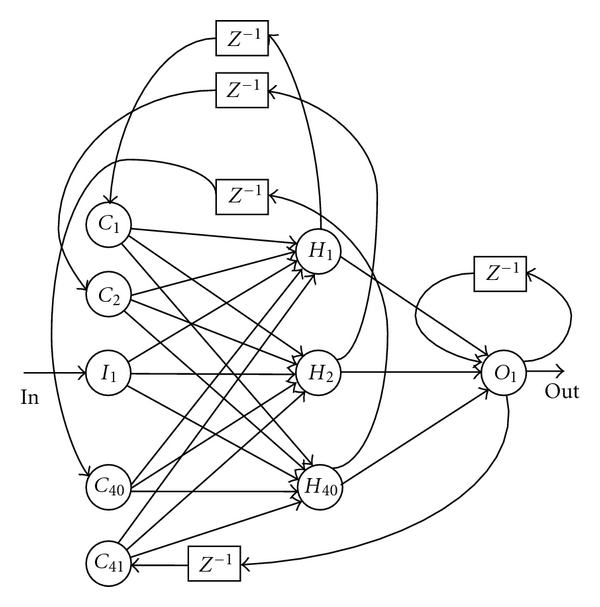
A structure of the trained Elman-Jordan neural network.

In the Elman network (Figure 1) [3], the input layer has a recurrent connection with the hidden layer. Therefore, at each time step, the output values of the hidden units are copied to the input units, which store them and use them for the next time step. This process allows the network to memorize some information from the past, in such a way to detect periodicity of the patterns in a better manner. Such capability can be exploited in our problem to recognize temporal patterns in the examined speech signals. The Jordan networks [4] are similar to the Elman networks. The context layer is, however, fed from the output layer instead of the hidden layer. To accelerate a learning (training) process of the Elman neural network, we propose a modified structure of the network. We combine the Elman network with the Jordan network and add another feedback for an output neuron as it is shown in Figure 2.

The pure Elman network consists of four layers:

- an input layer (in our model: the neuron *I*_1_),
- a hidden layer (in our model: the neurons *H*_1_, *H*_2_,…, *H*_40_),
- a context layer (in our model: the neurons *C*_1_, *C*_2_,…, *C*_40_),
- an output layer (in our model: the neuron *O*_1_).

*z* ^−1^ is a unit delay here.

To improve some learning ability of the pure Elman networks, we propose to add additional feedbacks in network structures. Experiments described in Section 5 validate this endeavor. We create (see Figure 2);

- feedback between an output layer and a hidden layer through the context neuron (in our model: the neuron *C*_41_), such feedback is used in the Jordan networks,
- feedback for an output layer.

A new network structure will be called the modified Elman-Jordan network. 

The Elman network, according to its structure, can store an internal state of a network. There can be values of signals of a hidden layer in time unit *t* − 1. Data are stored in the memory context. Because of storing values of a hidden layer for *t* − 1 we can make prediction for the next time unit for a given input value. In the case of learning neural networks with different architectures, we can distinguish three ways for making prediction for *x*(*t* + *s*), where *s* > 1:

1. training a network on values  *x*(*t*), *x*(*t* − 1), *x*(*t* − 1),….
2. training a network on each value *x*(*t* + *i*), where 1 ≥ *i* ≥ *s*. this way manifests good results for small *s*;
3. training a network only on a value *x*(*t* + 1), going iteratively to *x*(*t* + *s*) for any *s*.

In our case, we have used method (2).

The Jordan network can be classified as one of variants of the NARMA (Nonlinear Autoregressive Moving Average) model [11], where a context layer stores an output value for *t* − 1. It is assumed that a network with this structure does not have a memory. It processes only a value taken previously from the output. In the NARMA model, a context layer operates as a subtractor for an input value.

If we pass a single value to the network input in a given time unit *t*, then the Elman network stores the copies of values from a hidden layer for *t* − 1 in a context layer. The size of a hidden layer does not depend on the size of an output layer. In the case of the Jordan network, an output value for *t* − 1 is passed to a context layer. Therefore, the size of this layer depends on the size of an output layer. If a network has only one input and one output, then we have only one neuron in the context layer. In comparison with the Elman network, the Jordan network learns slower and requires a bigger structure. Therefore, the pure Jordan network cannot be used in solving our problem. In the modified Elman-Jordan network proposed by us, the network has feedbacks between selected layers. We provide additional information for the hidden layer. The hidden layer has an access to an input value, previous values of the hidden layer as well as an output value. Additional information has a big impact on modifying weights of the hidden layer. It leads to shortening a learning process and decreasing a network structure compared to the classical Elman network.

## 5. Experiments: Procedure and Results

Articulation is an individual patient feature. Therefore, we cannot train a neural network on the independent patterns of phonation of individual vowels. For each patient, a recorded speech signal is used for both training and testing of a neural network. The procedure is as follows. We divide the speech signal of an examined patient into time windows corresponding to phonemes. Next, we select randomly a number of time windows. This set of selected windows is used for determining some coefficient characterizing deformations in the speech signal. This coefficient is constituted by an error obtained during a testing stage of the neural network. We propose to use the approach similar to the cross-validation strategy. One time window is taken for training the neural network and the remaining ones for testing of the neural network. The network learns a selected time window. If the remaining windows are similar to the selected one in terms of the time patterns, then, for such windows, an error generated by the network in a testing stage is small. If significant replication disturbances in time appear for patients with the larynx disease, then an error generated by the network is greater. In this case, the time pattern is not preserved in the whole signal. Therefore, the error generated by the network reflects nonnatural disturbances in the patient phonation. Our approach can be expressed formally as it is shown in Algorithm 1. In the algorithm, we use the following functions (procedures):

- *Div2Win *(*S*): dividing the speech signal *S* into time windows corresponding to phonemes,
- *SelWin *(*W*): selecting randomly a number of time windows from the whole set *W*,
- *Train *(*N*, *w*): training a neural network *N* on a given time window *w*,
- *Test *(*N*, *w*): testing a neural network *N* on a given time window *w*,
- *MSE *(*E*): calculating a mean squared error for the absolute error vector *E*:
  (1) $$\text{MSE}\begin{array}{c} \left(E\right)=\frac{1}{n}\overset{n}{\underset{i=1}{\sum }}{\left(E_{i}\right)}^{2}, \end{array}$$
  where *n* is a number of elements in the vector *E*, *E*_*i*_ = *y*(*x*_*i*_) − *z*(*x*_*i*_) and *y*(*x*_*i*_) is the obtained output for *x*_*i*_, whereas *z*(*x*_*i*_) is the desired output for *x*_*i*_.
- *Avg *(*E*): calculating an arithmetic average for the vector *E* of errors.

In the experiments, sound samples were analyzed. The experiments were carried out on two groups of patients [2]. The first group included patients without disturbances of phonation—the control group (CG). They were confirmed by phoniatrist opinion. All patients were nonsmoking, so they did not have contact with toxic substances which can have an influence on the physiological state of vocal folds. The second group included patients of Otolaryngology Clinic of the Medical University of Lublin in Poland. They had clinically confirmed dysphonia as a result of Reinke's edema (RE) or laryngeal polyp (LP). The information about diseases was received from patients' documentations.

Experiments were carried out by a course of breathing exercises with instruction about a way of articulation. The task of all examined patients was to utter separately different Polish vowels with extended articulation as long as possible, without intonation and each on separate expiration. The microphone ECM-MS907 (Sony) was used for recording. Each sound sample was recorded on MiniDisc MZ-R55 (Sony). In MiniDisc, an analog signal is converted into a digital signal according to the CD (Compact Disc) standard (16 bits, 44.1 kHz), and next it is transformed by means of the ATRAC (Adaptive Transform Acoustic Coding for MiniDisc) system. A data size is reduced in the ratio of 5 to 1. The compression system is based on separating harmonics to which a human is most sensitive. Such harmonics are encoded with high precision. However, the less significant harmonics are encoded with the higher compression ratio. The MiniDisc can be used successfully. Effectiveness of such analysis was confirmed by Winholtz and Titze in 1998 [12].

**Figure 3:**
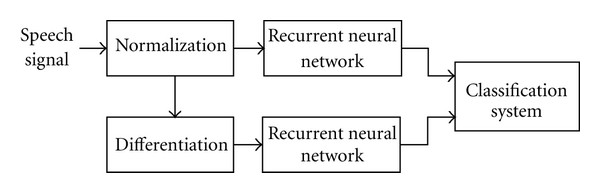
The block diagram of the process of the experiment.

The block diagram of the process of the experiment is shown in Figure 3.

Samples are normalized to the interval [0.0,1.0] before providing them to the next blocks. Next, the process of a speech signal analysis is divided into two paths. In the first path, the original signal (after normalization) is analyzed. In the second path, the derivative of the original signal (after normalization) is analyzed. It is well known from calculus that the derivative provides some additional information about the differentiated function, in our case, about the rate of change of the speech signal. This information can be useful in the classification process. After normalization, and alternatively differentiation, samples (as double numbers) are provided consecutively to the neural network inputs. Each patient can be located in two-dimensional space according to the average mean squared errors provided by RNNs for the original signal as well as for its derivative. In Table 1, we present results of experiments carried out using the pure Elman network. Next, we give exemplary results (see Table 2) obtained using the modified Elman-Jordan network described in Section 4. Both tables include results for women uttering vowel “A”. We give the average mean squared error ${\bar{E}}_{EN}$ (${\bar{E}}_{EJN}$) and the average number ${\bar{n}}_{EN}$ (${\bar{n}}_{EJN}$) of epochs needed to learn the network weights, respectively. The superscripts indicate

- CG: a woman from the control group,
- LP: a woman with laryngeal polyp,
- RE: a woman with Reinke's edema,

respectively.

It is easy to see that a combined structure of the Elman and Jordan neural networks improved a learning capability of the neural network while a distinction (between normal and disease states) capability remained at the same level. Sometimes, the Elman network is not capable to learn a given pattern with a number of epochs equal to 10000 (see, e.g., case *w*_2_^CG^ in Table 1). Observations made by us are very important for further research, especially in the context of a created computer tool for diagnosis of larynx diseases.

Patients described in two-dimensional space can be classified using different data mining and machine learning methods (see, e.g., [13]). We can use for the classification purpose methods embedded in well-known computer tools, among others, in

- the Rough Set Exploration System (RSES)—a software tool featuring a library of methods and a graphical user interface supporting a variety of rough set based computations [14];
- WEKA: a collection of machine learning algorithms for data mining tasks [15].

In the most generic format, medical diagnosis rules are conditional statements of the form: IF *conditions* (*symptoms*), THEN *decision* (*diagnosis*). The rule expresses the relationship between symptoms determined on the basis of examination and diagnosis which should be taken for these symptoms before the treatment. In our case, symptoms are determined on the basis of patient's speech signal analysis using RNNs. It is easy to see that making a distinction between laryngeal polyp and Reinke's edema on the basis of the proposed approach is, in fact, impossible. Therefore, this problem will be considered separately in the future. Now, each patient can be classified into two categories:

- *no*: patient without laryngopathy,
- *yes*: patient with laryngopathy.

For the input data to be classified (which are used to learn or extract relationships between data), we have a tabular form (see example in Table 3) which is formally called a decision system (decision table) *S* = (*U*, *A*, *d*) in the Pawlak's form [16]. *U* is a set of cases (patients), *A* is a set of descriptive attributes, and *d* is a decision attribute determining a category. In our case, *A* = {*a*_1_, *a*_2_}, where *a*_1_ is the attribute corresponding to the average mean squared error provided by RNNs for the original signal and *a*_2_ is the attribute corresponding to the average mean squared error provided by RNNs for the differentiated signal. Moreover, *d* denotes the existence of laryngopathy.

Values of descriptive attributes (*a*_1_ and *a*_2_) can be treated as continuous quantitative data. Building classification rules for such data can be difficult and/or highly inefficient. Therefore, for some rule generation algorithms, the so-called discretization is a necessary preprocessing step [13]. Its overall goal is to reduce the number of values by grouping them into a number of intervals. In many cases, discretization enables obtaining a higher quality of classification rules. Some discretization techniques based on rough sets and Boolean reasoning have been presented in [17]. On the other hand, some algorithms (especially based on decision trees) applied for continuous data lead to rules with conditions in the form of intervals.

In our experiments, we have used, for example, two different approaches to rule generation:

- the direct method: the LEM2 algorithm included, among others, in the RSES system;
- the decision tree based method: the J4.8 algorithm, included, among others, in the WEKA system.

The first algorithm is based on a covering approach. The LEM2 algorithm was proposed by J. Grzymala-Busse in [18]. Covering-based algorithms produce less rules than algorithms based on an explicit reduct calculation. J4.8 is WEKA's improved implementation of the C4.5 algorithm. C4.5 is an algorithm used to generate a decision tree developed by R. Quinlan [19]. C4.5 builds decision trees from a set of training data using the concept of information entropy. The LEM2 algorithm produces (in case of using the modified Elman-Jordan network) two rules for analyzed data:

- IF *a*_1_ ∈ (0.0326, *∞*), THEN *d* = *yes*,
- IF *a*_1_ ∈ (−*∞*, 0.0326) AND *a*_2_ ∈ (−*∞*, 0.0695), THEN *d* = *no*.

For the training set, a classification error is 0%. One case is not covered by any generated rule.

**Figure 4:**
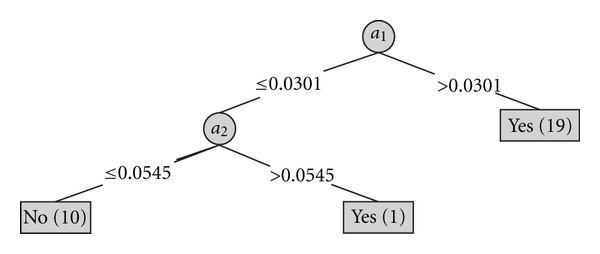
A decision tree obtained using the J4.8 algorithm.

The similar rules are produced through the J4.8 algorithm. A decision tree obtained using the J4.8 algorithm has the form shown in Figure 4. The rules read from a decision tree have the following form (each rule is generated by making a conjunction of all the tests encountered on the path from the root to the leaf):

- IF *a*_1_ ∈ (0.0301, *∞*), THEN *d* = *yes*,
- IF *a*_1_ ∈ (−*∞*, 0.0301] AND *a*_2_ ∈ (−*∞*, 0.0545], THEN *d* = *no*,
- IF *a*_1_ ∈ (−*∞*, 0.0301] AND *a*_2_ ∈ (0.0545, *∞*), THEN *d* = *yes*.

A number in brackets, for each decision tree node, denotes a number of cases classified into the category assigned to this node. For the training set, a classification error is 0%.

Exemplary results show that patients described in two-dimensional (two attributes corresponding to the average mean squared errors provided by RNNs for the original signal and the differentiated signal, resp.) space can easily be discriminated between normal and disease states.

## 6. Conclusions

The following matters can be noticed on the basis of experiments described in the paper.

- Combining and modifying the structures of two recurrent neural networks (the Elman network with the Jordan network) used for assessment of speech signal deformations for patients with larynx diseases leads to improving a learning capability of the neural network, while a distinction (between normal and disease states) capability remains at the same level. Such an acceleration is important if a diagnostic decision should be made in real time.
- The proposed approach based on analysis of speech signals using recurrent neural networks can be a preliminary step in making a distinction between normal and disease states.

We can list the following main problems which will be considered in the future:

- hybridization of classification methods of patients with laryngopathies, where the approach presented in this paper constitutes one of elements (beside frequency based and time-frequency-based approaches).
- designing methods enabling distinction between different larynx diseases (e.g., laryngeal polyp and Reinke's edema). The approach presented in this paper does not enable us to make this distinction;
- automation of the process of dividing the speech signal into time windows corresponding to phonemes (a single window is limited by peaks). At the current stage, the windowing method is not automatic. A part of samples corresponding phonemes is strongly noised, especially at the beginning and at the end, and this part cannot be provided to the neural network input.

The presented results will be helpful for selection of suitable techniques for a created computer tool supporting diagnosis of larynx diseases.

## Figures and Tables

**Algorithm 1:**
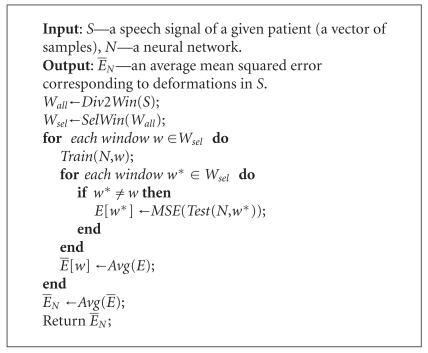
Algorithm for calculating an average mean squared error corresponding to deformations in a speech signal.

**Table 1:** Selected results of experiments for women obtained using the Elman network.

Patient ID	Original signal		Ddifferentiated signal	
	${\bar{E}}_{EN}$	${\bar{n}}_{EN}$	${\bar{E}}_{EN}$	${\bar{n}}_{EN}$

*w*_1_^CG^	0.0068	389	0.0245	455
*w*_2_^CG^	2.4523	335	0.0208	650
*w*_3_^CG^	0.017	501	0.0341	497
*w*_4_^CG^	0.0109	597	0.01	422
*w*_5_^CG^	0.0332	662	0.0566	650
*w*_6_^CG^	0.0178	609	0.0324	656
*w*_7_^CG^	0.0096	428	0.0202	333
*w*_8_^CG^	0.0068	318	0.028	575
*w*_9_^CG^	0.008	490	0.0216	925
*w*_10_^CG^	0.0084	553	0.05	504

*w*_1_^LP^	0.172	331	0.1081	564
*w*_2_^LP^	0.2764	536	0.1936	622
*w*_3_^LP^	0.0518	566	0.0533	593
*w*_4_^LP^	0.0268	504	0.0879	498
*w*_5_^LP^	0.0418	646	0.1726	547
*w*_6_^LP^	0.2107	444	0.2468	506
*w*_7_^LP^	0.0921	1040	0.1687	439
*w*_8_^LP^	0.0364	992	0.1396	758
*w*_9_^LP^	0.038	541	0.1061	826
*w*_10_^LP^	0.1461	363	0.2448	711

*w*_1_^RE^	0.039	360	0.055	487
*w*_2_^RE^	0.1006	452	0.1	729
*w*_3_^RE^	0.1021	446	0.1583	608
*w*_4_^RE^	0.0636	780	0.0804	586
*w*_5_^RE^	0.1626	446	0.2376	545
*w*_6_^RE^	0.1953	477	0.1905	500
*w*_7_^RE^	0.2027	337	0.1661	378
*w*_8_^RE^	0.1927	457	0.1367	717
*w*_9_^RE^	0.2908	939	0.2139	865
*w*_10_^RE^	0.4357	679	0.3795	820

**Table 2:** Selected results of experiments for women obtained using the modified Elman-Jordan network.

Patient ID	Original signal		Ddifferentiated signal	
	${\bar{E}}_{EN}$	${\bar{n}}_{EN}$	${\bar{E}}_{EN}$	${\bar{n}}_{EN}$

*w*_1_^CG^	0.0061	88	0.0228	103
*w*_2_^CG^	0.0111	92	0.0193	90
*w*_3_^CG^	0.0178	107	0.0347	117
*w*_4_^CG^	0.0115	96	0.0086	35
*w*_5_^CG^	0.0301	146	0.0537	123
*w*_6_^CG^	0.0166	104	0.0328	76
*w*_7_^CG^	0.0086	78	0.0201	178
*w*_8_^CG^	0.0068	108	0.0248	116
*w*_9_^CG^	0.008	162	0.0204	106
*w*_10_^CG^	0.0087	119	0.0494	76

*w*_1_^LP^	0.1677	92	0.1042	204
*w*_2_^LP^	0.3107	191	0.2108	47
*w*_3_^LP^	0.0542	96	0.0545	97
*w*_4_^LP^	0.0258	142	0.0853	144
*w*_5_^LP^	0.0423	239	0.1716	119
*w*_6_^LP^	0.2134	71	0.2428	86
*w*_7_^LP^	0.0877	40	0.1648	109
*w*_8_^LP^	0.0351	72	0.1362	132
*w*_9_^LP^	0.037	180	0.105	123
*w*_10_^LP^	0.1411	160	0.2382	96

*w*_1_^RE^	0.0395	148	0.0534	117
*w*_2_^RE^	0.097	99	0.0991	96
*w*_3_^RE^	0.1053	115	0.1583	117
*w*_4_^RE^	0.0628	36	0.0784	70
*w*_5_^RE^	0.1596	133	0.2332	116
*w*_6_^RE^	0.1951	95	0.1945	90
*w*_7_^RE^	0.1954	51	0.1669	177
*w*_8_^RE^	0.191	99	0.1358	120
*w*_9_^RE^	0.281	106	0.2084	100
*w*_10_^RE^	0.4366	65	0.3746	77

**Table 3:** The input data to be classified (fragment).

Patient ID	*a*_1_	*a*_2_	*d*
*w*_1^CG	0.0061	0.0228	* no *
*w*_2^CG	0.0111	0.0193	* no *
⋮	⋮	⋮	⋮
*w*_1_^LP^	0.1677	0.1042	*ye* *s*
*w*_2_^LP^	0.3107	0.2108	*ye* *s*
⋮	⋮	⋮	⋮
*w*_1^RE	0.0395	0.0534	* yes *
*w*_2^RE	0.097	0.0991	* yes *
⋮	⋮	⋮	⋮
